# Instrumental assessment of physiotherapy and onabolulinumtoxin-A on cervical and headache parameters in chronic migraine

**DOI:** 10.1007/s10072-021-05491-w

**Published:** 2021-08-05

**Authors:** Manuela Deodato, Antonio Granato, Caterina Borgino, Alessandra Galmonte, Paolo Manganotti

**Affiliations:** 1 Department of Medical, Surgical and Health Sciences, University of Trieste, 34100 Trieste, Italy; 2 Department of Life Sciences, University of Trieste, 34100 Trieste, Italy; 3 Azienda Sanitaria Universitaria Giuliano Isontina (ASUGI), 34128 Trieste, Italy

**Keywords:** Chronic migraine, Cervical range of motion, Forward head posture, Onabolulinumtoxin-A, Physiotherapy

## Abstract

**Introduction:**

The purpose of the present study is to compare the effect of the physiotherapy to onabolulinumtoxin-A, and their combination, in relation to cervical and headache parameters in patients with chronic migraine.

**Methods:**

This is an observational cohort study conducted by a headache center and a physiotherapy degree course on 30 patients with chronic migraine. The patients were distributed in three groups of treatments for three months: onabolulinumtoxin-A only, physiotherapy only, and onabolulinumtoxin-A plus physiotherapy. The patients were evaluated, before and after each treatment, using the following: the postural assessment software SAPO for the forward head posture; the CROM goniometer for the cervical range of motion; the Migraine Disability Assessment Score for headache parameters.

**Results:**

After 3 months of each treatment, the scores obtained for the headache-related disability and the frequency of migraine decreased significantly for all groups, but the pain intensity scores changed significantly only in the onabolulinumtoxin-A (*p* = 0.01) and in the onabolulinumtoxin-A plus physiotherapy groups (*p* = 0.007). On the other hand, the forward head posture was reduced significantly in the physiotherapy (*p* = 0.002) and in the onabolulinumtoxin-A plus physiotherapy groups (*p* = 0.003). The cervical range of motion increased significantly in certain directions in the physiotherapy group and in the onabolulinumtoxin-A plus physiotherapy groups.

**Conclusions:**

The physiotherapy improved the cervical parameters. The onabolulinumtoxin-A decreased pain intensity. As a consequence, it can be said that the combined treatment was more useful than a mono-therapy alone. From our results, it can be concluded that onabolulinumtoxin-A plus physiotherapy could be a good option in the management of chronic migraine.

**Supplementary Information:**

The online version contains supplementary material available at 10.1007/s10072-021-05491-w.

## Introduction

Headache disorders were one of the three leading causes of disability for both sexes in 2017 [1]. A primary concern of headache is migraine that represents one of the most severe headache in terms of pain intensity and of headache related disability. Therefore, a rehabilitative challenge is to find multi-professional new approaches for a more effective management of migraine [2].

The present study focuses on chronic migraine. It is based on a considerable amount of literature that highlights the key aspect of musculoskeletal dysfunctions in the trigeminal sensitization and, consequently, in the increase of frequency and intensity of migraine [2–6]. These musculoskeletal dysfunctions are present not only in the trigeminal area, but throughout the spine. As a result, migraine patients suffer from symptoms such as neck and low back pain; forward head posture (FHP); pericranial tenderness; active trigger points; and reduction in cervical/dorsal mobility [7–10]. Especially, FHP is associated with an increase of anterior and posterior tension force in the neck that could lead to a decrease in the cervical range of motion (CROM), neck pain, and dynamic muscle imbalance [6, 11–13]. Nevertheless, up to now, there has been no detailed investigation of the effects of different treatments on these musculoskeletal dysfunctions.

Today, onabotulinumtoxin-A is one of the main pharmacological prophylactic choice for chronic migraine [14–16]. As regards non-pharmacological treatments, physiotherapy is also helpful in primary headaches, and it increases the effectiveness of pharmacological treatments [17, 18]. The injection sites of onabotulinumtoxin-A often coincide with the areas treated in physiotherapy; that is why a decrease in cranio-cervical hypersensitivity may reduce sensitization in the trigeminal area, even if through different mechanisms. However, no studies have been conducted previously that compare the effects of these two kinds of therapies on chronic migraine, nor the possible added value of their combination.

The first aim of the present study is to compare the efficacy of physiotherapy and onabotulinumtoxin-A in chronic migraine on cervical parameters, such as FHP and CROM, and on headache parameters, such as headache-related disability, the migraine frequency, and intensity. The second aim is to evaluate the effect of the combined treatment onabotulinumtoxin-A plus physiotherapy on the above-mentioned parameters.

## Methods

An observational cohort study was performed on 3 groups of patients with chronic migraine. The study was approved by the institutional review board and was conducted in accordance with the Declaration of Helsinki. The first step was the enrollment visit (T0, baseline) at the headache center with the following criteria of inclusion: diagnosis of chronic migraine [19]; lack of success of at least 3 previous prophylactic treatments; and age over 18. On the other hand, exclusion criteria were as follows: pregnancy; serious psychiatric pathologies; serious pathologies such as traumas, tumors, or infections; significant surgical procedures during the previous 12 months; physiotherapy or other prophylactic treatment in the previous 3 months; and patients with cervical spine diseases. Thereafter, each participant was given a diary to record frequency, intensity, and duration of attacks.

Following a 1-month period (T1), all diaries were examined, the eligibility criterion being a headache frequency ≥15 days per month. After that, data concerning FHP, CROM, and headache parameters were collected, before and after each treatment. The bio-photogrammetric evaluation was performed using the postural assessment software PAS/SAPO (version 0.69). SAPO was selected for its high intra- and inter-operator reliability, validity, and repeatability [20, 21]. Thus, the FHP was assessed with the cranio-vertebral angle (C7 and the ear tragus). The setting and the equipment were as follows: a camera (Nikon Coolpix L100), placed on a tripod at 1.63 m and at 3.45 m of distance from the patients, and 3 adhesive white spherical markers (15-mm diameter) applied to the patients’ skin (one on each tragus, and one on the C7 spinous process). Two photos were taken on the sagittal (right and left) plane of the patient in a standing position.

The CROM inclinometer is one of the more practical methods for measuring the active CROM [22]. During the evaluation, patients were asked to sit on a chair with ankles, knees, and hips at 90° and feet resting firmly on the ground. The CROM was repeated actively in all directions by the patients twice; the average of the two measurements was considered for the data analysis.

The MIDAS questionnaire is particularly useful in studying the impact of migraine [23]. MIDAS consists of 5 questions that assess the burden of migraine expressed in days, in the following items: absence from work or school; inability to carry out household chores; and to take part in family, social, or leisure activities. Furthermore, other 2 sections, MIDAS-A and MIDAS-B, were used to assess, respectively, migraine frequency (number of days with migraine), and the average of pain intensity (on a scale ranging from 1 to 10).

After the first data collection, the cohort was divided into the 3 groups, according to the patient’s choice and the specialist’s opinion as per normal clinical practice: physical therapy only (PT) (mono-therapy), onabotulinumtoxin-A only (BoNT-A) (mono-therapy), and onabotulinumtoxin-A plus physical therapy (BoNT-A+PT) (combined therapy). All patients were naïve about onabolulinumtoxin-A and physiotherapy.

The patients were allowed to take symptomatic medications in case of severe headache, according to the international guidelines [24]. However, the patients were only asked to limit the consumption to twice per week and to note the dosage and the frequency of use in their diary.

### Onabotulinumtoxin-A only protocol

A specific FAD-approved protocol PREEMPT [14–16] was used for the onabotulinumtoxin-A only and the onabotulinumtoxin-A plus physical therapy groups. The PREEMPT protocol consists of 31 infiltrations of 5 units of onabotulinumtoxin-A carried out using a small insulin needle, into seven specific head and neck muscles sites bilaterally (i.e., frontalis (20 U 4 sites), corrugator (10 U 2 sites), procerus (5 U 1 site), occipitalis (30 U 6 sites), temporalis (40 U 8 sites), trapezius (30 U 6 sites), and cervical paraspinal muscle group (20 U 4 sites)), for a total of 155 units. In order to identify more precisely the muscles, the infiltrations were guided by the electromyography and applied by 2 expert neurologists in a single 40-min long session.

### Physiotherapy only protocol

The physiotherapy only protocol was an integrated treatment [25, 26] of manual therapy (30 min) and active exercises (30 min). It was organized in 15 one-hour long individual sessions, and it was carried out by 2 expert physiotherapists. The sessions were scheduled weekly and performed over the 3 months of the study.

#### Manual therapy

The manual therapy techniques were chosen from the areas most treated in the recent studies, such as sacral region; dorsal and cervical spine; diaphragm; and cranial region. The treatment started with soft mobilizations in the sacral region [9]. The second step consisted in the manual treatment of the diaphragm [27]. Next, central posterior-anterior mobilizations were applied to the cervico-dorsal area at spinous processes from C2 to D12 segment [8, 10, 18]. Finally, soft tissue mobilizations were included on the sub-occipital muscles and on the epicranial aponeurosis [28, 29].

#### Active exercises

A progression of active exercises was selected with a predefined goal: improving endurance of trunk functions in sitting position. The protocol started in supine “Hook-Lying” position for the first five sessions, the next five sessions in sitting position, and the last five sessions in sitting position on a balance board. Patients were asked to maintain their trunk in a vertical position during head and upper and lower limb movements while avoiding FHP [30, 31].

#### Onabotulinumtoxin-A + physiotherapy protocol

The onabotulinumtoxin-A plus physiotherapy group underwent first the onabotulinumtoxin-A protocol and, after 4 days, the physiotherapy protocol.

The final evaluation was performed (T2) after 3 months of each of the 3 kinds of treatment with the same outcomes: FHP; CROM; and the MIDAS, MIDAS-A, and MIDAS-B questionnaires.

### Data analysis

GraphPad InStat 3.06 was used for data analysis: the Wilcoxon non-parametric test, for the initial and final evaluations, and the Kruskal-Wallis test (Nonparametric ANOVA), for the variance among groups. The statistical significance level was ɑ 95% (0.05). The GraphPad Prism 8.4.1 (676) was chosen for the graphic representation of the data.

## Results

A total of 30 patients were enrolled, 10 for each group, with diagnosis of chronic migraine based on the diagnostic criteria of ICDH3-beta: “Headache occurring on 15 or more days/month for more than 3 months, which, on at least 8 days/month, has the features of migraine headache.” The physiotherapy (PT) only group consisted of 10 women, mean age of 51.1 years (SD 15.7) with 20.5 (SD 5.4) days of migraine per month; the onabotulinumtoxin-A (BoNT-A) only group consisted of 2 men and 8 women, mean age of 52.7 years (SD 12.7) with 22.4 (SD 6.5) days of migraine per month; and the onabotulinumtoxin-A plus physiotherapy (BoNT-A+PT) group consisted of 2 men and 8 women, mean age of 51.6 years (SD 12.8) with 22.5 (SD 6.4) days of migraine per month. The medications intake per month were 22.8 (SD 7) for BoNT-A, 25.3 (SD 11.4) for BoNT-A+PT, and 22.9 (SD 11.5) for PT. No patient was on medication over use. No statistical differences were registered among the 3 groups at T1 in terms of age (*p* = 0.9); CVA (*p* = 0.7); range of motion (flexion *p* = 0.5; extension *p* = 0.2; right lateral flexion *p* = 0.7; left lateral flexion *p* = 0.6; right rotation *p* = 0.18; left rotation *p* = 0.7); MIDAS score (*p* = 0.9); MIDAS-A (*p* = 0.5); MIDAS-B (*p* = 0.1); medication intake per month (*p* = 0.9); and frequency of migraine at baseline (*p* = 0.6).

### Cervical parameters

The first set of questions was aimed to evaluate the clinical change in the cervical spine after the 3 different types of treatments in patients with chronic migraine. Table 1 presents an overview of all the cervical parameters before and after each treatment.

**Table 1:** Cervical parameters in the three groups: onabotulinumtoxin-A (BoNT-A); onabotulinumtoxin-A + physiotherapy (BoNT-A+PT); and physiotherapy (PT)

**Cervical parameters**	**BoNT-A**	**BoNT-A+PT**	**PT**
Forward head posture	T1 138.5° (SD±7.5)	T1 135.2° (SD±3.5)	T1 136.4° (SD±3.9)
	T2 138.9° (SD±5.8)	T2 132.7° (SD±3.6)**	T2 131.1° (SD±3)**
Flexion	T1 42.3° (SD±13.8)	T1 42.2° (SD±8.2)	T1 38° (SD±9.3)
	T2 42.3° (SD±5.5)	T2 56.5° (SD±9.9)**	T2 49.4° (SD±13)*
Extension	T1 40.7° (SD±7.8)	T1 43.1° (SD±15)	T1 48.3° (SD±8.5)
	T2 37.9° (SD±12)	T2 51.8° (SD±12.6)*	T2 57° (SD±10.1)**
Lateral flexion right	T1 30.4° (SD±11.9)	T1 27.7° (SD±4.4)	T1 29.5° (SD±10.2)
	T2 27.6° (SD±10.8)	T2 37.4° (SD±3)**	T2 36.2° (SD±9.9)**
Lateral flexion left	T1 31.8° (SD±9.4)	T1 28.7° (SD±7.4)	T1 29.9° (SD±10.9)
	T2 27.6° (SD±10.8)	T2 36.1° (SD±3.2)**	T2 37° (SD±10.7)**
Rotation right	T1 55.1° (SD±6.4)	T1 60.5° (SD±5.8)	T1 59.9° (SD±11.6)
	T2 54.9° (SD±7)	T2 57.2° (SD±17.2)	T2 64.9° (SD±13.6)*
Rotation left	T1 54.8° (SD±5.7)	T1 56.6° (SD±7.9)	T1 54.4° (SD±14.3)
	T2 55.9° (SD±7.5)	T2 54.6° (SD±15.1)	T2 57.6° (SD±15.2)

*p < 0.05; **p < 0.01 Wilcoxon non-parametric test at the fist evaluation (T1) and at the end of each treatment (T2): *BoNT-A* onabotulinumtoxin-A, *BoNT-A+PT* onabotulinumtoxin-A plus physiotherapy, *PT* physiotherapy

#### FHP

**Fig. 1:**
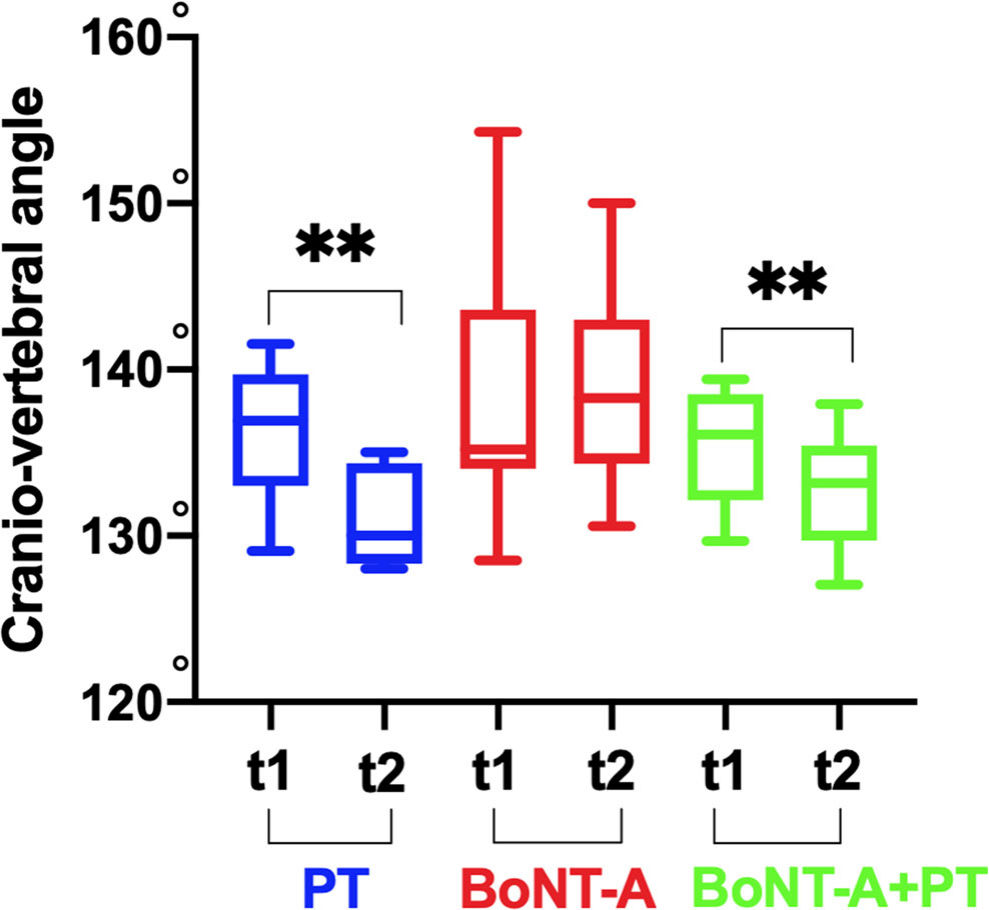
Forward head posture (FHP). FHP in onabotulinumtoxin-A only (BoNT-A), onabotulinumtoxin-A plus physiotherapy (BoNT-A+PT), and physiotherapy only (PT) at the first evaluation (T1) and at the end of each treatments (T2); Wilcoxon non-parametric test *p < 0.05; **p < 0.01

At T2 assessment, the improvement of the FHP was statistically significant for both the PT only (*p* = 0.002; CIs95% +2.1/+8.3) and the BoNT-A+PT (*p* = 0.003; CIs95% +1.4/+3.4) groups, but no for the BoNT-A only group. Moreover, the Kruskal-Wallis test revealed statistically significant differences among groups (*p* = 0.005): PT vs. BoNT-A -12.4 **p ≤ 0.01; BoNT-A vs. BoNT-A+PT -8.9 ns *p* ≥ 0.05; PT vs. BoNT-A+PT -3.5 ns *p* ≥ 0.05. In Figure 1, there is a clear decreasing trend of the FHP for both the PT only and in the BoNT-A+PT groups.

#### CROM

**Fig. 2:**
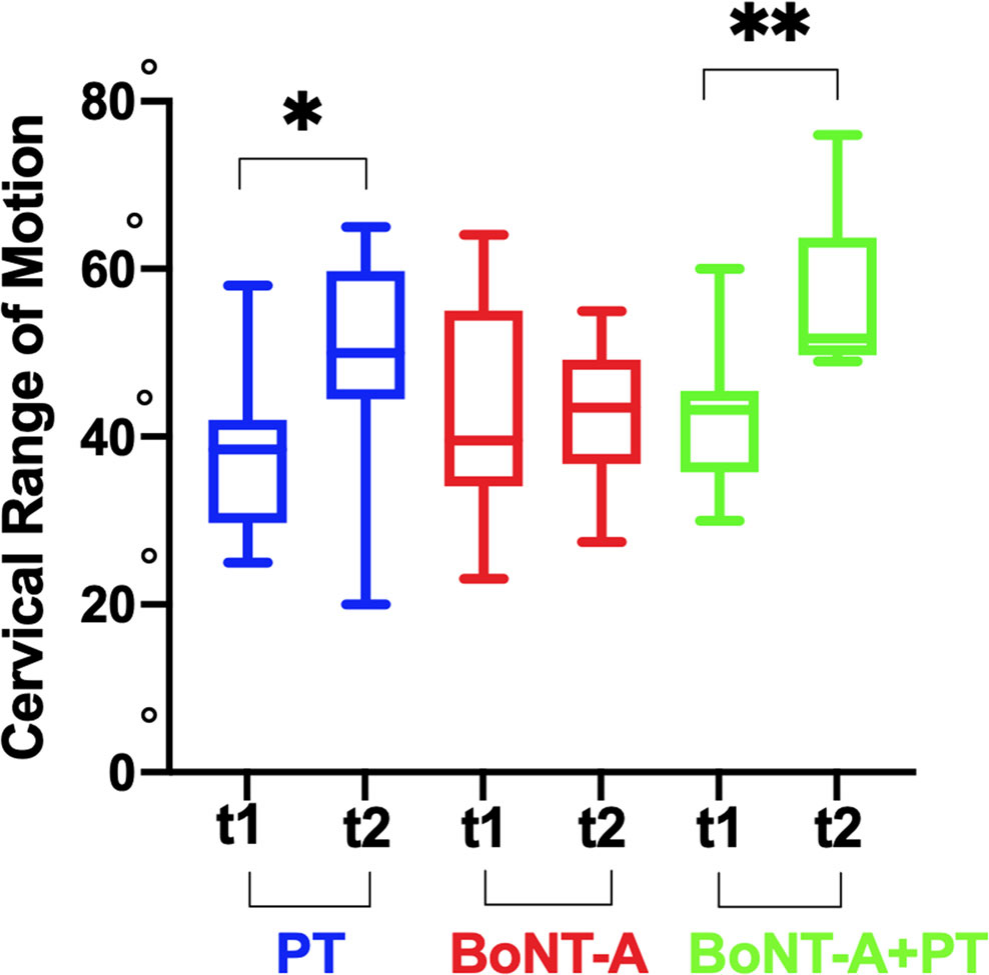
Cervical range of motion (CROM): flexion in onabotulinumtoxin-A only (BoNT-A), onabotulinumtoxin-A plus physiotherapy (BoNT-A+PT), and physiotherapy only (PT) at the first evaluation (T1) and at the end of each treatments (T2); Wilcoxon non-parametric test *p < 0.05; **p < 0.01

**Fig. 3:**
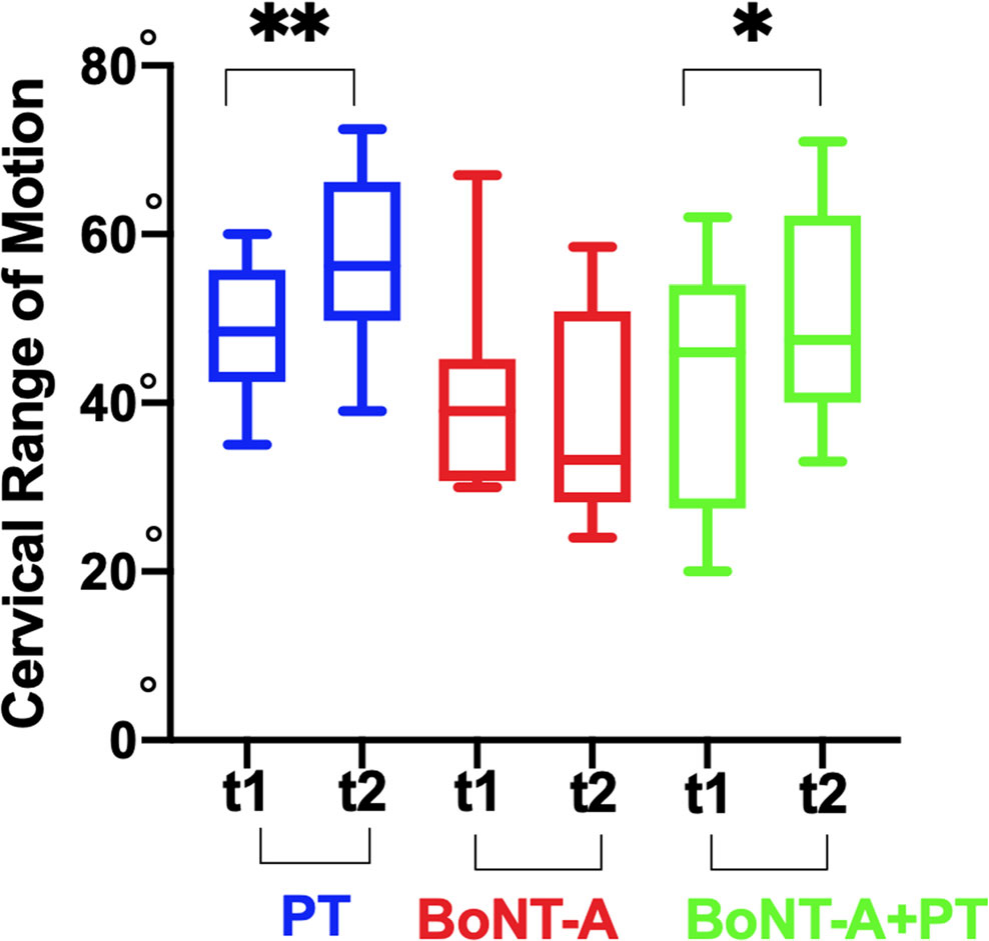
Cervical range of motion (CROM): extension in onabotulinumtoxin-A only (BoNT-A), onabotulinumtoxin-A plus physiotherapy (BoNT-A+PT), and physiotherapy only (PT) at the first evaluation (T1) and at the end of each treatments (T2); Wilcoxon non-parametric test *p < 0.05; **p < 0.01

As regards the flexion and the extension, the PT only group and the BoNT-A+PT group showed a statistically significant increase in both directions (flexion in PT *p* = 0.03 CIs95% -22.1/-0.5; flexion in BoNT-A+PT *p* = 0.002 CIs95% -23/-5.6; extension in PT *p* = 0.002 CIs95% -13.6/-3.8; extension in BoNT-A+PT *p* = 0.04 CIs95% -17.3/-0.1). Furthermore, the variance among the groups was statistically significant both for the flexion *p* = 0.009 (PT vs. BoNT-A 7.250 ns *p* > 0.05; PT vs. BoNT-A+PT -4.55 ns *p* > 0.05; BoNT-A+PT vs. BoNT-A -11.8** *p* < 0.01) and for extension *p* = 0.009 (PT vs. BoNT-A 11.4* *p* < 0.05; PT vs. BoNT-A+PT 2.8 ns *p* > 0.05; BoNT-A vs. BoNT-A+PT -8.6 ns *p* > 0.05) (Figures 2 and 3).

Concerning the lateral flexion, it was enhanced only in two groups: PT only group and BoNT-A+PT group both on the left and on the right directions (lateral flexion left PT *p* = 0.003 CIs95% -10/-3; lateral flexion right PT *p* = 0.003 CIs95% -10.9/-2.3; lateral flexion left BoNT-A+PT *p* = 0.009 CIs95% -12.1/-2.7; lateral flexion right BoNT-A+PT *p* = 0.002 CIs95% -12.2/-7.1). In addition to this, the difference among groups was significant in lateral flexion right *p* = 0.03 (PT vs. BoNT-A 7.85 ns *p* > 0.05; PT vs. BoNT-A+PT -1.55 ns *p* > 0.05; BoNT-A vs. BoNT-A+PT 9.4 ns *p* > 0.05), but not quite significant (*p* = 0.07) in lateral flexion left.

As shown in Table 1, in the results about rotation, there was a statistically significant improvement only for the right side in the PT only group (*p* = 0.01; ICs95% -9/-02), but no differences among the 3 groups were found (left *p* = 0.7; right *p* = 0.3).

### MIDAS

The second set of analyses examined the impact of the 3 different treatments on the headache-related disability, frequency, and intensity of migraine attacks. Table 2 provides the results obtained from the analysis performed on the headache parameters before and after each of the 3 kinds of treatment.

**Table 2:** Headache parameters: Migraine Disability Assessment Score (MIDAS), MIDAS-A, and MIDAS-B and diary in all groups: onabotulinumtoxin-A (BoNT-A); onabotulinumtoxin-A + physiotherapy (BoNT-A+PT); and physiotherapy (PT)

**Headache parameters**	**BoNT-A**	**BoNT-A+PT**	**PT**
MIDAS	T1 84.9 (SD±38)	T1 84.5 (SD±50.3)	T1 81.7 (SD±52)
	T2 41.6 (SD±35.6)*	T2 48.8 (SD±53.6)*	T2 45.6 (SD±38.7)*
MIDAS-A	T1 63.8 (SD±16.9)	T1 70.9 (SD±20.6)	T1 66.7 (SD±18.5)
	T2 39.1 (SD±17.6)**	T2 50.6 (SD±28.8)**	T2 43 (SD±24.1)**
MIDAS-B	T1 7.7 (SD±0.8)	T1 7.6 (SD±0.5)	T1 6.8 (SD±1.3)
	T2 5.7 (SD±1.6)*	T2 5.6 (SD±1.6)**	T2 5.8 (SD±1.3)
Frequency	T1 22.4 (SD±6.5)	T1 22.5 (SD±6.4)	T1 20.5 (SD±5.4)
	T2 15.1 (SD±7.5)*	T2 15 (SD±9.1)*	T2 13.3 (SD±7)*

*p < 0.05; **p < 0.01 Wilcoxon non-parametric test at the fist evaluation (T1) and at the end of each treatment (T2): *BoNT-A* onabotulinumtoxin-A, *BoNT-A+PT* onabotulinumtoxin-A plus physiotherapy, *PT* physiotherapy. MIDAS, the number of days of absence from work or school, inability to carry out household chores, and to take part in family, social, or leisure activities; MIDAS-A, number of days with migraine in the last 3 months; MIDAS-B, the average of pain intensity on a scale ranging from 1 to 10; frequency, number of days with migraine per month

Table 2 reports a statistically significant reduction in the MIDAS score for all the groups [BoNT-A only (*p* = 0.01; ICs95% +15.5/+71), BoNT-A+PT (*p* = 0.04; ICs95% -1.2/+72.6), and PT only (*p* = 0.01; ICs95% +11.7/+60.4)], but not among groups (*p* = 0.9).

**Fig. 4:**
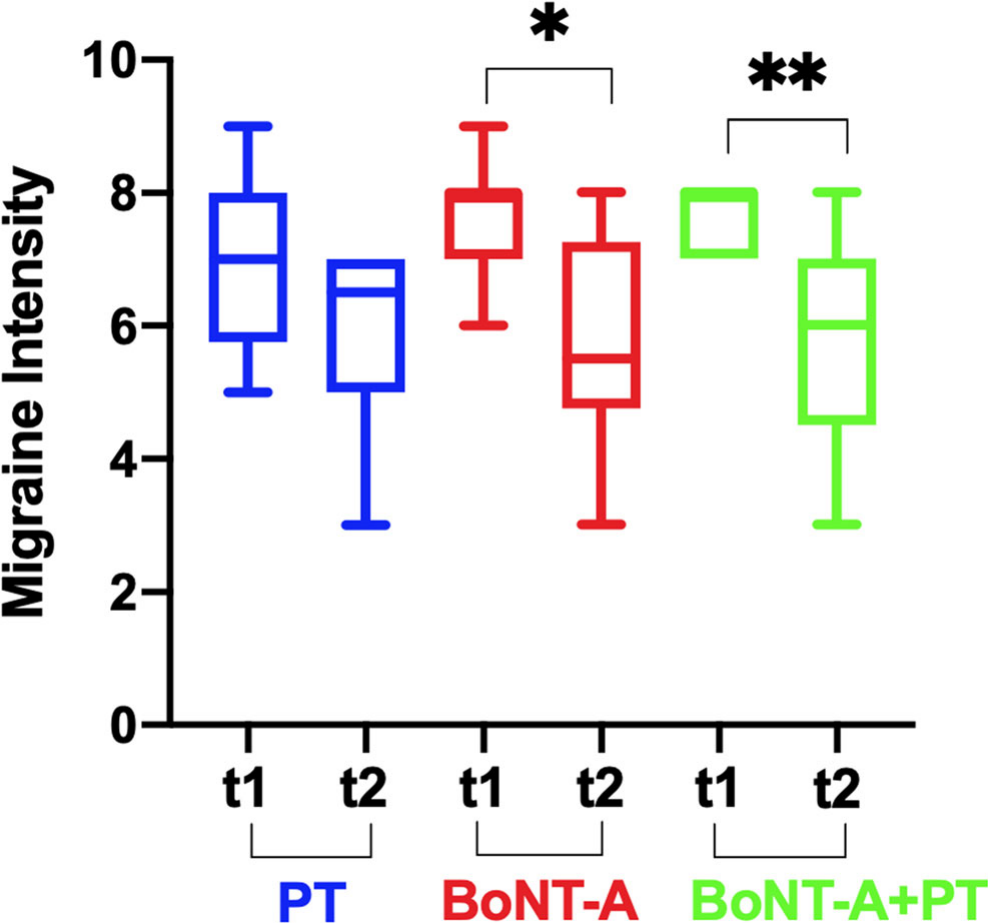
Migraine Disability Assessment Score (MIDAS): MIDAS-B intensity of headache attack (on a scale of 1–10) in the previous 3 months, in onabotulinumtoxin-A only (BoNT-A), onabotulinumtoxin-A plus physiotherapy (BoNT-A+PT), and physiotherapy only (PT) at the first evaluation (T1) and at the end of each treatments (T2); Wilcoxon non-parametric test *p < 0.05; **p < 0.01

In addition, the MIDAS-A frequency decreased significantly for all the groups: for the PT only group (*p* = 0.02; ICs95% +8.2/+39.1); for the BoNT-A+PT group (*p* = 0.007; ICs95% +9.4/+31.1); and for the BoNT-A only group (*p* = 0.002; ICs95% +7.9/+41.4). On the other hand, we found a statistically significant difference in the MIDAS-B only for the BoNT-A only (*p* = 0.01; ICs95% +0.8/+3.1) and in the BoNT-A+PT groups (*p* = 0.007; ICs95% +0.8/+3.1), but not among the three groups (MIDAS-A *p* = 0.7; MIDAS-B; *p* = 0.9) (Table 2) (Fig. 4).

## Discussion

Prior studies have described the importance of peripheral mechanisms in the sensitization of the trigeminal-cervical complex and consequently in migraine transmission [6–8, 13]. The present study for the first time has investigated, in patients with chronic migraine, the effect on both cervical and headache parameters of 3 different types of treatments, that are as follows: (1) onabolulinumtoxin-A only; (2) physiotherapy integrated protocol (i.e., manual therapy and active exercise) only; and (3) onabolulinumtoxin-A plus physiotherapy. The first remarkable finding of the present research was that the FHP and the CROM improved significantly for the physiotherapy (PT) only group, but not for the onabolulinumtoxin-A only (BoNT-A) group. On the other hand, we found that the intensity of migraine decreased significantly for the BoNT- A only group, but not for the PT only group. Finally, the most clinically relevant finding was that the onabolulinumtoxin-A plus physiotherapy (BoNT-A+PT) group reported a statistically significant positive change for both the cervical as well as for all the measured headache parameters.

No previous research in patients with chronic migraine had investigated the FHP and the CROM parameters variation after BoNT-A or PT treatments. Nevertheless, the different results for the FHP and the CROM we obtained support previous research concerning the temporary adverse events of BoNT-A: neck pain/muscular weakness in the sites of BoNT-A’s injections and musculoskeletal stiffness in the nearby sites. This imbalance between muscular stiffness and weakness could potentially lead to temporary FHP and CROM limitations [32, 33]. Our study did not find any improvement in the cervical parameters after the BoNT-A only treatment. These results are also in line with the hypothesis that individualized injections sites could inactivate the myofascial trigger points and improve the cervical outcomes better than the fixed sites specified in the PREEMPT protocol [34]. In addition, it seems that BoNT-A have more efficacy in migraine than in cervicogenic or tension type headache [6, 35], where the cervical spine represents the major trigger. Moreover, the improvement of the cervical parameters in both the PT groups (i.e., PT only and BoNT-A+PT) seems to be consistent with the literature that recommends the use of manual therapy in chronic migraine for the musculoskeletal dysfunctions [8, 17, 18]. Hence, we hypothesize that the temporary adverse events of BoNT-A may be limited by the addition of PT.

Concerning headache parameters, the results of the present research show that the headache related disability and frequency improved for both the PT and the BoNT-A groups, but the intensity of migraine improved only when the BoNT-A was administered. These results support the suggestion that PT is effective in reducing frequency and duration, but a quite limited evidence about its efficacy in reducing pain intensity [30].

The BoNT-A+PT group showed an improvement for cervical as well as for headache parameters, according to the idea that migraine responds better to combined treatments [36]. Moreover, our study results further support the evidence found in previous researches that observed that the combined treatment BoNT-A+PT is more effective than a mono-therapy in pelvic pain [37], upper limb spasticity [38], and chronic facial synkinesis [39].

As regards the limitations of the present work, the most relevant ones are as follows: the consumption of symptomatic medication, the small study population, and the absence of a long follow-up. The consumption of the symptomatic medications may be an uncontrolled variable, but the guidelines allow patients to take symptomatic medications in case of severe headache. Therefore, the only possibility we had was to set a limit to the consumption of this medications to twice a week. Next, the small sample does not allow a statistical interpretation of gender variability, but explains the casual finding of the significant of some parameters but not of others (right vs. left). Finally, long follow-up is important to understand if early findings are maintained or improved over time. In fact, many studies addressed the importance of repetitive cycles over 1 year of onabotulinumtoxin-A, while in our 3-month study, the effects of a single injection can be evaluated. Despite that, our study presents 3 main relevant novelties: (1) it analyzes the effects of 2 treatments (BoNT-A and PT), both singularly and in combination, in relation to both cervical and headache parameters; (2) it is one of the few studies involving PT with a specific and integrated treatment; and (3) the cervical parameters were assessed with an instrumental objective evaluation method.

The findings of our study incorporate a number of practice implications. First, the pharmacological and the non-pharmacological treatments produce an important desensitization effect on the trigeminal area, through different mechanisms. The BoNT-A only treatment would seem to be more useful in relieving pain intensity through the inhibition of the neurogenic inflammation [14–16], while the PT, with manual therapy, would seem to be more useful in posture correction through a decrease of tissue contraction in the upper cervical spine and in pericranial areas [29]. In addition, the PT, with active exercise, may reduce the frequency and the duration of attacks due to an increase of neuromodulators levels in plasma [30]. Consequently, the most important clinical implication is that the best approach for patients with chronic migraine could be the use of the BoNT-A in combination with an integrated PT treatment (manual therapy and active exercise) [25, 36]. Second, the combined treatment BoNT-A+PT could decrease the cervical temporary adverse effects of the BoNT-A; it is demonstrated by the differences in the FHP measurements with SAPO and in CROM before and after the 3 treatments. It can therefore be assumed that if long-term treatment of BoNT-A has resulted in better benefits and prolonged efficacy in patients [40], the repetitive cycles of BoNT-A+PT could lead to better outcomes in terms of frequency, intensity, and duration of the pain but also in terms of cervical parameters. In this way, repetitive cycles could be scheduled over a year that include the following: four injections of BoNT-A, administered every 12 weeks, combined with four cycles of PT of 15 sessions each.

## Conclusions

To summarize, this combined multi-professional approach is potentially very useful for both the patients and the health systems, because it may offer a more complete clinical management of the outcomes related to the complex multifactorial disorder that chronic migraine represents. A large randomized controlled trial could provide better evidence concerning the correlation between the cervical parameters and the headache parameters; moreover, it would be interesting to know if early finds are maintained or improved over time.

## Supplementary Information

ESM 1 (PNG 47 kb)

High resolution image (TIFF 97 kb)

ESM 2 (PNG 43 kb)

High resolution image (TIFF 92 kb)

## Data Availability

The principal author takes full responsibility for the data presented in this study, analysis of the data, conclusions, and conduct of the research. The datasets page containing authors’ details analyzed during the current study are available from the corresponding author on reasonable request.

## Abbreviations

PT: Physiotherapy
BoNT-A: Onabolulinumtoxin-A
FHP: Forward head posture
CROM: Cervical range of motion
MIDAS: Migraine Disability Assessment Score

## References

[1] Global, regional, and national incidence, prevalence, and years lived with disability for 354 diseases and injuries for 195 countries and territories, 1990–2017: a systematic analysis for the Global Burden of Disease Study 2017 - The Lancet. https://www.thelancet.com/journals/lancet/article/PIIS0140-6736(18)32279-7/fulltext. Access 10.1016/S0140-6736(18)32279-7 PMC6227754 30496104

[2] Charles A (2018). The pathophysiology of migraine: implications for clinical management. Lancet Neurol.

[3] Fusco M, D’Andrea G, Miccichè F (2003). Neurogenic inflammation in primary headaches. Neurol Sci.

[4] Aguggia M, Saracco MG (2010). Pathophysiology of migraine chronification. Neurol Sci.

[5] Panerai AE (2013). Is migraine a disorder of the central nervous system?. Neurol Sci.

[6] Blumenfeld A, Siavoshi S (2018). The challenges of cervicogenic headache. Curr Pain Headache Rep.

[7] Mongini F, Rota E, Deregibus A, Mura F, Francia Germani A, Mongini T (2005). A comparative analysis of personality profile and muscle tenderness between chronic migraine and chronic tension-type headache. Neurol Sci.

[8] Luedtke K, Starke W, May A (2018). Musculoskeletal dysfunction in migraine patients. Cephalalgia.

[9] Ashina S, Lipton RB, Bendtsen L, Hajiyeva N, Buse DC, Lyngberg AC, Jensen R (2018). Increased pain sensitivity in migraine and tension-type headache coexistent with low back pain: a cross-sectional population study. Eur J Pain.

[10] Liang Z, Galea O, Thomas L, Jull G, Treleaven J (2019). Cervical musculoskeletal impairments in migraine and tension type headache: a systematic review and meta-analysis. Musculoskelet Sci Pract.

[11] Ferracini GN, Chaves TC, Dach F, Bevilaqua-Grossi D, Fernández-de-las-Peñas C, Speciali JG (2016). Relationship between active trigger points and head/neck posture in patients with migraine. Am J Phys Med Rehabil.

[12] Ferracini GN, Chaves TC, Dach F, Bevilaqua-Grossi D, Fernández-de-las-Peñas C, Speciali JG (2017). Analysis of the cranio-cervical curvatures in subjects with migraine with and without neck pain. Physiotherapy.

[13] Mingels S, Dankaerts W, Granitzer M (2019). Is there support for the paradigm ‘spinal posture as a trigger for episodic headache’? A comprehensive review. Curr Pain Headache Rep.

[14] Blumenfeld A, Silberstein SD, Dodick DW, Aurora SK, Turkel CC, Binder WJ (2010). Method of injection of onabotulinumtoxinA for chronic migraine: a safe, well-tolerated, and effective treatment paradigm based on the PREEMPT clinical program. Headache.

[15] Bendtsen L, Sacco S, Ashina M, Mitsikostas D, Ahmed F, Pozo-Rosich P, Martelletti P (2018). Guideline on the use of onabotulinumtoxinA in chronic migraine: a consensus statement from the European Headache Federation. The Journal of Headache and Pain.

[16] Dodick DW, Turkel CC, DeGryse RE (2010). OnabotulinumtoxinA for treatment of chronic migraine: pooled results from the double-blind, randomized, placebo-controlled phases of the PREEMPT clinical program. Headache.

[17] Fernández-de-Las-Peñas C, Cuadrado ML (2016). Physical therapy for headaches. Cephalalgia.

[18] Moore CS, Sibbritt DW, Adams J (2017). A critical review of manual therapy use for headache disorders: prevalence, profiles, motivations, communication and self-reported effectiveness. BMC Neurol.

[19] (2018) Headache Classification Committee of the International Headache Society (IHS) The International Classification of Headache Disorders, 3rd edition. Cephalalgia 38:1–211. 10.1177/0333102417738202 10.1177/0333102417738202 29368949

[20] Krawczky B, Pacheco AG, Mainenti MRM (2014). A systematic review of the angular values obtained by computerized photogrammetry in sagittal plane: a proposal for reference values. J Manipulative Physiol Ther.

[21] Macedo Ribeiro AF, Bergmann A, Lemos T, Pacheco AG, Mello Russo M, Santos de Oliveira LA, de Carvalho Rodrigues E (2017). Reference values for human posture measurements based on computerized photogrammetry: a systematic review. J Manipulative Physiol Ther.

[22] Alahmari K, Reddy RS, Silvian P, Ahmad I, Nagaraj V, Mahtab M (2017). Intra- and inter-rater reliability of neutral head position and target head position tests in patients with and without neck pain. Brazilian Journal of Physical Therapy.

[23] Peng K-P, Wang S-J (2012). Migraine diagnosis: screening items, instruments, and scales. Acta Anaesthesiol Taiwan.

[24] Tassorelli C, Diener H-C, Dodick DW, Silberstein SD, Lipton RB, Ashina M, Becker WJ, Ferrari MD, Goadsby PJ, Pozo-Rosich P, Wang SJ, for the International Headache Society Clinical Trials Standing Committee (2018). Guidelines of the International Headache Society for controlled trials of preventive treatment of chronic migraine in adults. Cephalalgia.

[25] Gaul C, Liesering-Latta E, Schäfer B, Fritsche G, Holle D (2016). Integrated multidisciplinary care of headache disorders: a narrative review. Cephalalgia.

[26] Grazzi L (2013). Multidisciplinary approach to patients with chronic migraine and medication overuse: experience at the Besta Headache Center. Neurol Sci.

[27] Jafari H, Courtois I, Van den Bergh O (2017). Pain and respiration: a systematic review. Pain.

[28] Castien RF, van der Wouden JC, De Hertogh W (2018). Pressure pain thresholds over the cranio-cervical region in headache: a systematic review and meta-analysis. The Journal of Headache and Pain.

[29] Deodato M, Guolo F, Monticco A, Fornari M, Manganotti P, Granato A (2019). Osteopathic manipulative therapy in patients with chronic tension-type headache: a pilot study. J Am Osteopath Assoc..

[30] Lemmens J, De Pauw J, Van Soom T (2019). The effect of aerobic exercise on the number of migraine days, duration and pain intensity in migraine: a systematic literature review and meta-analysis. J Headache Pain.

[31] Terrin A, Mainardi F, Zanchin G, Maggioni F (2019). Sports, physical activity and headache in the classical age: historical descriptions from the first sports textbook, “De arte gymnastica”, by Girolamo Mercuriale. Neurol Sci.

[32] Diener H-C, Dodick DW, Turkel CC, Demos G, DeGryse RE, Earl NL, Brin MF (2014). Pooled analysis of the safety and tolerability of onabotulinumtoxinA in the treatment of chronic migraine. Eur J Neurol.

[33] Matharu M, Pascual J, Nilsson Remahl I, Straube A, Lum A, Davar G, Odom D, Bennett L, Proctor C, Gutierrez L, Andrews E, Johannes C (2017). Utilization and safety of onabotulinumtoxinA for the prophylactic treatment of chronic migraine from an observational study in Europe. Cephalalgia.

[34] Ranoux D, Martiné G, Espagne-Dubreuilh G, Amilhaud-Bordier M, Caire F, Magy L (2017). OnabotulinumtoxinA injections in chronic migraine, targeted to sites of pericranial myofascial pain: an observational, open label, real-life cohort study. J Headache Pain.

[35] Becker WJ, Chitsantikul P (2011). Cervicogenic headache and onabotulinumtoxinA: where do we stand?. Cephalalgia.

[36] Ghanbari A, Askarzadeh S, Petramfar P, Mohamadi M (2015). Migraine responds better to a combination of medical therapy and trigger point management than routine medical therapy alone. NeuroRehabilitation.

[37] Halder GE, Scott L, Wyman A, Mora N, Miladinovic B, Bassaly R, Hoyte L (2017). Botox combined with myofascial release physical therapy as a treatment for myofascial pelvic pain. Investig Clin Urol.

[38] Lannin NA, Ada L, English C, Ratcliffe J, Faux SG, Palit M, Gonzalez S, Olver J, Cameron I, Crotty M, Bowman M, Milte R, Vratsistas-Curto A, McNamara A, Shiner C, Lynch E, Schneider E, Beaumont L, Killington M, Coulter M, Sindhusake D, Anthonisz B, Mei Khor H, Tan J, Teo K, Ng L, Huang L, Paul M, Simon N, Gupta N, Martens R, Bolitho S, Morrison S, Hooper S, Chow Y, Watanabe Y, Cowling A, Flu C, Edwards D, Toma E, Hendrey G, Sheehan J, Butler J, Hocking J, Rutzou L, White M, Snigg M, Hughes R, Sweeney S, Flint S, Levy T, Bramah V, Lathlean C, McCallum C, Chui E, Allan F, Webber H, Cameron J, Campbell J, Lawson J, Zenouith K, Borschmann K, Moloney K, Jolliffe L, Cameron L, Howlett O, Nicks R, O’Keefe S, on behalf of the InTENSE Trial Group (2020). Effect of additional rehabilitation after botulinum toxin-A on upper limb activity in chronic stroke: the InTENSE Trial. Stroke.

[39] Mandrini S, Comelli M, Dall’angelo A (2016). Long-term facial improvement after repeated BoNT-A injections and mirror biofeedback exercises for chronic facial synkinesis: a case-series study. Eur J Phys Rehabil Med.

[40] Guerzoni S, Pellesi L, Baraldi C, Cainazzo MM, Negro A, Martelletti P, Pini LA (2017) Long-term treatment benefits and prolonged efficacy of onabotulinumtoxinA in patients affected by chronic migraine and medication overuse headache over 3 years of therapy. Front Neurol 8. 8. 10.3389/fneur.2017.00586 10.3389/fneur.2017.00586 PMC5676047 29163347

