# Supplementary figures and images for: Instrumental assessment of physiotherapy and onabolulinumtoxin-A on cervical and headache parameters in chronic migraine
Source: Neurol Sci. 2021 Aug 5;43(3):2021–9. doi: 10.1007/s10072-021-05491-w (PMC8860953; doi:10.1007/s10072-021-05491-w)

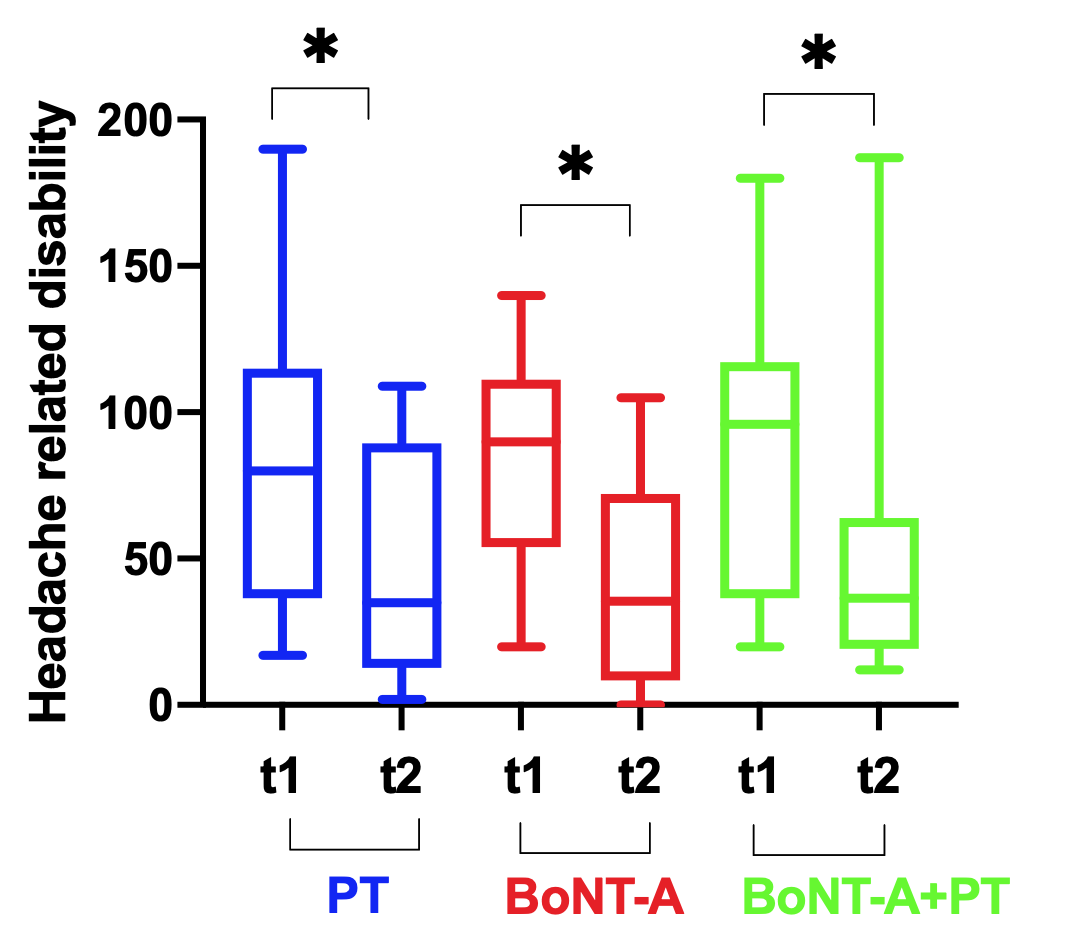

Supplement: Supplementary file 1 — (PNG 47 kb) [file 10072_2021_5491_Fig5_ESM.png]

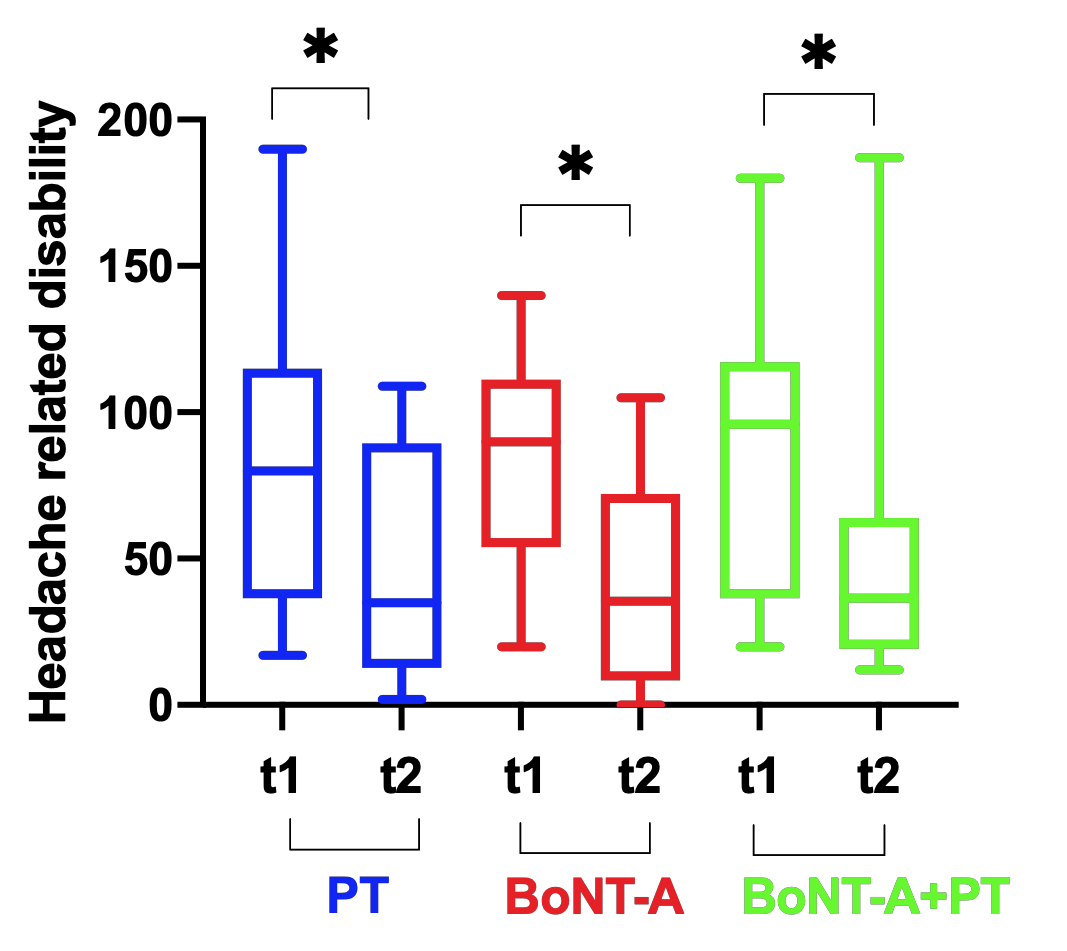

Supplement: Supplementary file 2 — High resolution image (TIFF 97 kb) [file 10072_2021_5491_MOESM1_ESM.tiff]

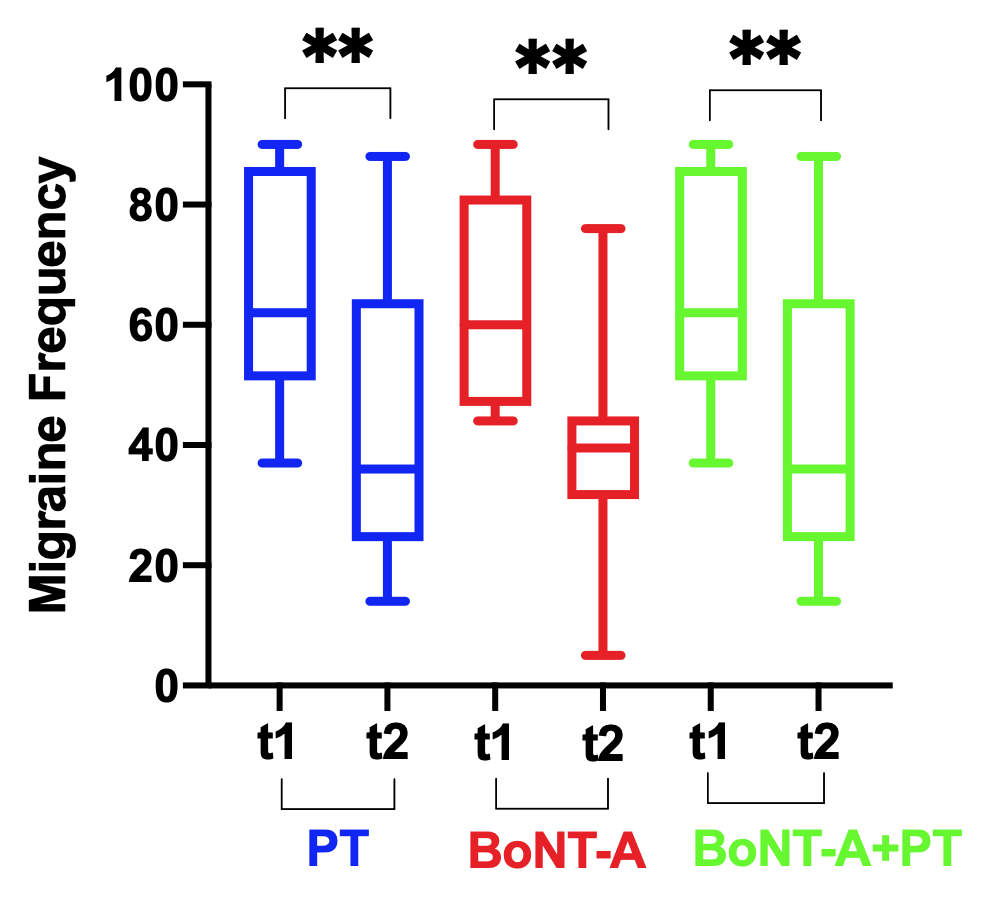

Supplement: Supplementary file 3 — (PNG 43 kb) [file 10072_2021_5491_Fig6_ESM.png]

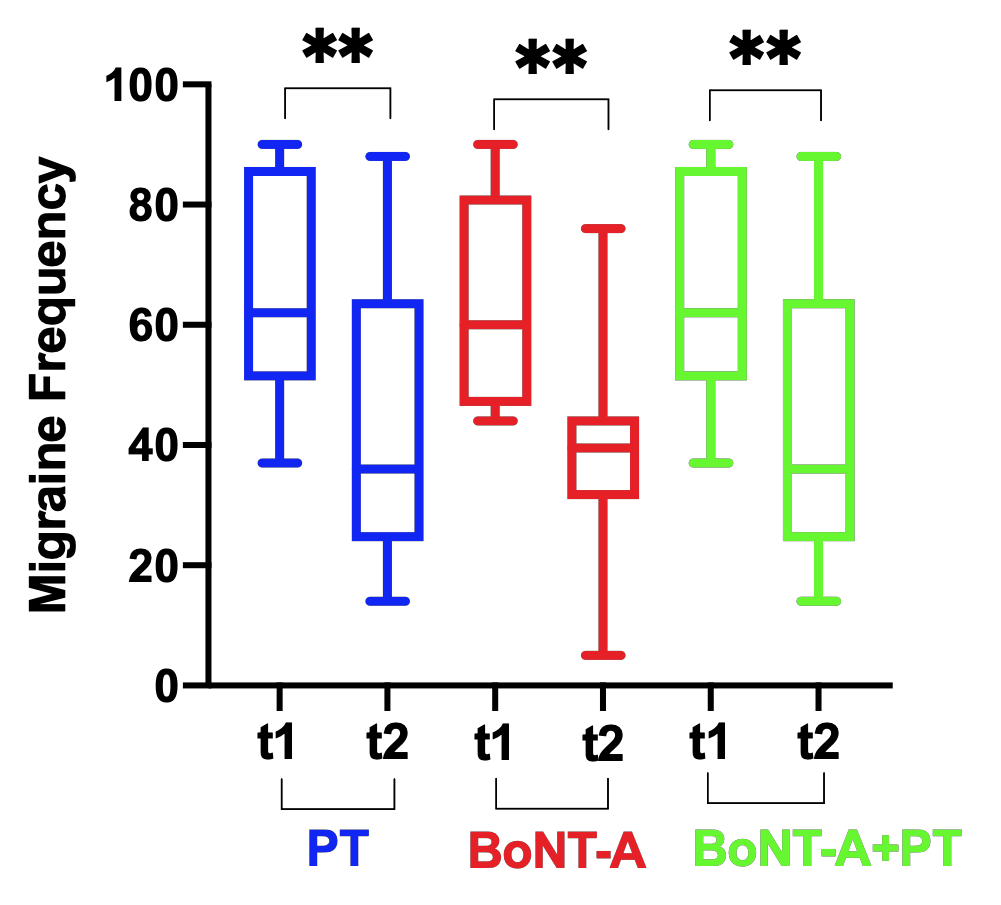

Supplement: Supplementary file 4 — High resolution image (TIFF 92 kb) [file 10072_2021_5491_MOESM2_ESM.tiff]
